# Association between compound dietary antioxidant index and all-cause and cancer mortality in patients with chronic obstructive pulmonary disease: results from NHANES 1999–2018

**DOI:** 10.3389/fmed.2025.1544841

**Published:** 2025-03-21

**Authors:** Wenqiang Li, Jingshan Bai, Yanlei Ge, Yuting Fan, Qian Huang, Zhiping Deng

**Affiliations:** ^1^Department of Pulmonary and Critical Care Medicine, Zigong First People's Hospital, Zigong, China; ^2^Department of Respiratory Medicine, Xiongan Xuanwu Hospital, Xiong'an, China; ^3^Department of Respiratory Medicine, North China University of Science and Technology Affiliated Hospital, Tangshan, China; ^4^Department of Clinical Medicine, North Sichuan Medical College, Nanchong, China; ^5^Department of Respiratory Medicine, Dazhou Dachuan District People's Hospital (Dazhou Third People's Hospital), Dazhou, China

**Keywords:** COPD, CDAI, all-cause mortality, cancer mortality, NHANES

## Abstract

**Objective:**

Chronic obstructive pulmonary disease (COPD) is one of the most important causes of death in the world, and its core is chronic inflammation. Antioxidants play a positive role in the onset and prognosis of chronic respiratory diseases. In maintaining human health, the composite dietary antioxidant index (CDAI) plays an important function. Therefore, the purpose of the current study was to investigate the relationship between CDAI and all-cause and cancer mortality in individuals with COPD.

**Methods:**

A prospective cohort study was conducted by investigating NHANES data between 1999–2018. The study included people who satisfied the inclusion and exclusion criteria. In this study, the association between CDAI and all-cause and cancer mortality was investigated using weighted Cox regression. The relationship between them is illustrated by drawing constrained cubic spline curves (RCS). Finally, subgroup analysis is used to further verify.

**Results:**

The study included 1,534 participants. CDAI was associated with COPD patients mortality, and after adjusting for multiple factors, we observed a 5% reduction in the risk of all-cause mortality (HR = 0.95, 95% CI: 0.92–0.97) was associated with a 9% lower risk of cancer mortality for each one-unit increase in CDAI (HR = 0.91, 95% CI: 0.85–0.98). After adjusting for multiple factors, high CDAI was associated with a reduced risk of mortality, with patients in the high CDAI group having 35% lower all-cause mortality than those in the low CDAI group (*HR* = 0.65, 95% *CI*: 0.50–0.85), the high CDAI group had a 61% lower risk of cancer mortality (*HR* = 0.39,95% *CI*: 0.23–0.68). Subgroup analysis and sensitivity analysis showed a consistent association between CDAI and COPD mortality.

**Conclusion:**

Our study highlights the inverse association between CDAI and all-cause and cancer mortality in patients with COPD. Further prospective studies are needed to confirm the role of CDAI in mortality risk in patients with COPD.

## 1 Introduction

Chronic obstructive pulmonary disease (COPD) is characterized by progressive, irreversible decline in lung function and persistent airflow limitation. It is the third leading cause of disability-adjusted life years (DALYs) globally (1). The prevalence of COPD is approximately 10.3% among individuals aged 30 to 79 years (1). COPD is characterized by chronic inflammation that begins in the airways and, as the disease progresses, can gradually affect other organs, eventually becoming a systemic condition (2). COPD primarily affects individuals over the age of 40. As people age and are exposed to harmful substances such as tobacco smoke, the risk of developing various malignancies, including lung cancer, increases (3). Given the limited effectiveness of pharmacological treatments for COPD, it is essential to identify modifiable lifestyle factors that can mitigate the risk of developing the disease (4).

Recent research highlights reactive oxygen species (ROS) as crucial in oxidative stress. Overproduction of ROS is linked to chronic inflammation, COPD, and various malignancies. Under physiological conditions, antioxidants typically regulate ROS levels. However, antioxidants can be obtained both *in vivo* and ex vivo (5). Malnutrition and an antioxidant deficit might intensify the body’s reaction to oxidative stress molecules, causing tissue damage (6). The Composite Dietary Antioxidant Index (CDAI) is a novel measure that aggregates various antioxidants, including vitamins, minerals, and phytochemicals, to reflect the overall antioxidant capacity of the diet (7). This index aims to provide a comprehensive view of dietary antioxidant intake, crucial for understanding its potential health benefits. As these antioxidants are incorporated, the CDAI value increases, indicating a higher antioxidant capacity of the diet. The composite dietary antioxidant index (CDAI) is a valid method for evaluating an individual’s antioxidant consumption. It also concentrates on carotenoids including zinc, selenium, and vitamins A, C, and E (8). A higher CDAI score reflects greater antioxidant capacity.

Currently, a substantial body of research utilizes the CDAI to analyze the risk relationships between antioxidant-rich diets and common diseases (9). Although existing studies suggest that certain dietary nutrients can prevent airway inflammation in the general population, research on the relationship between CDAI and mortality in COPD patients is lacking. To investigate the relationship between them, we used community population data from the National Health and Nutrition Examination Survey (NHANES).

## 2 Materials and methods

### 2.1 Data source and study design

We conducted a study using data from the Centers for Disease Control and Prevention’s 1999–2018 National Health and Nutrition Examination Survey. Based on changes to the updated Helsinki Declaration, the research protocol was authorized by the National Center for Health Statistics Research Ethics Review Board. Informed consent was provided by NHANES participants. A complete description of the study can be found at: https://www.cdc.gov/nchs/nhanes/index.htm.

**Figure 1:**
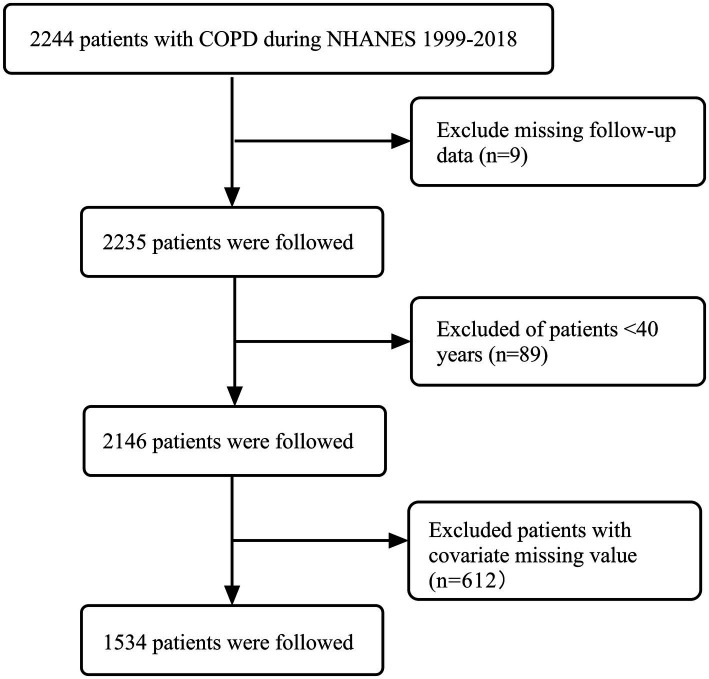
Flowchart of participant selection.

We selected a cohort of COPD patients (*n* = 2,244) from the 1999–2018 NHANES data. Exclusion criteria: (1) excluded missing follow-up data (*n* = 9); (2) excluded patients <40 years (*n* = 89); and (3) excluded patients with covariate missing value (*n* = 612). Finally, a total of 1,534 COPD patients were analyzed (Figure 1).

### 2.2 Outcome assessment

All-cause mortality was calculated by combining NHANES data with NDI records as of December 31, 2019. ICD-10 codes C00-C97 were used to reflect cancer fatalities.

### 2.3 Factors of exposure

The CDAI is predominantly affected by dietary factors. Intake of antioxidants, micronutrients, and total energy was calculated using the US Department of Agriculture’s Dietary Research Food and Nutrition Database (7). Using the questionnaire survey, we assessed each participant’s intake of dietary supplements in the past month, including dose, frequency, and number of doses. To calculate the CDAI, we standardized the intake of six specific dietary vitamins and minerals by subtracting their global average and dividing by the global standard deviation (10). We then computed the CDAI by summing the standardized intake of these vitamins and minerals as described below.


$$\mathrm{CDAI=}\overset{n=6}{\underset{i=1}{\sum }}\frac{\mathrm{Individual Intake}-\mathrm{Mean}}{SD}$$

### 2.4 Covariates

The relevant covariates were obtained from questionnaires. Age (40–64, ≥65), sex (male, female), race (white, other), health insurance (yes, no), educational level (<high school, high school diploma, >High school), poverty income ratio (PIR) (< 1.3, 1.3–3.5, > 3.5), body mass index (BMI) (< 25, 25–30, > 30), smoking status (never, fomer, now), alcohol consumption status (yes, no), hypertension (yes, no), diabetes (borderline, yes, no) and COPD (survival, death) were used as categorical variables. There are two categories for alcohol consumption status: never drank (that is, consuming less than 12 drinks in a lifetime) and drank (that is, consuming 12 or more drinks in a lifetime) (11). Smoking status is classified as never smokers (having smoked less than 100 cigarettes), past smokers (not smoking at the moment but having smoked more than 100 cigarettes), or current smokers (having smoked more than 100 cigarettes and smoking daily or sometimes) (12). The PIR is the family income divided by the relevant poverty threshold for the survey year (13). Participants self-reported their diabetes and hypertension diagnoses using questionnaires.

### 2.5 Statistical analyses

R version 4.3.3 was used for the statistical analyses. The intricate stratified sampling and sample weights were taken into consideration in all statistical studies. For categorical variables, differences between groups were analyzed using chi-square tests. The “maxstat” package’s maximum selected rank statistics method was used to determine the ideal CDAI cutoff point linked to survival outcomes (14, 15). Using weighted Cox regression analysis, the relationship between CDAI and cancer and all-cause death in individuals with COPD was evaluated. Model 1 adjusted for sex, age, and race; Model 2 further adjusted for education, smoking, poverty-income ratio (PIR), insurance, and alcohol consumption; Model 3 included additional adjustments for BMI, diabetes, and hypertension. To examine the association and interactions between CDAI and mortality, subgroup analyses were conducted based on age, sex, race, BMI, and smoking status. Furthermore, the possible dose–response association between CDAI and cancer and all-cause mortality in individuals with COPD was visualized using restricted cubic spline (RCS) models. Finally, by removing deaths that occurred within two years of the baseline evaluation, sensitivity analyses were performed to evaluate the reliability of the primary findings.

## 3 Results

### 3.1 Characteristics of the study population

**Figure 2:**
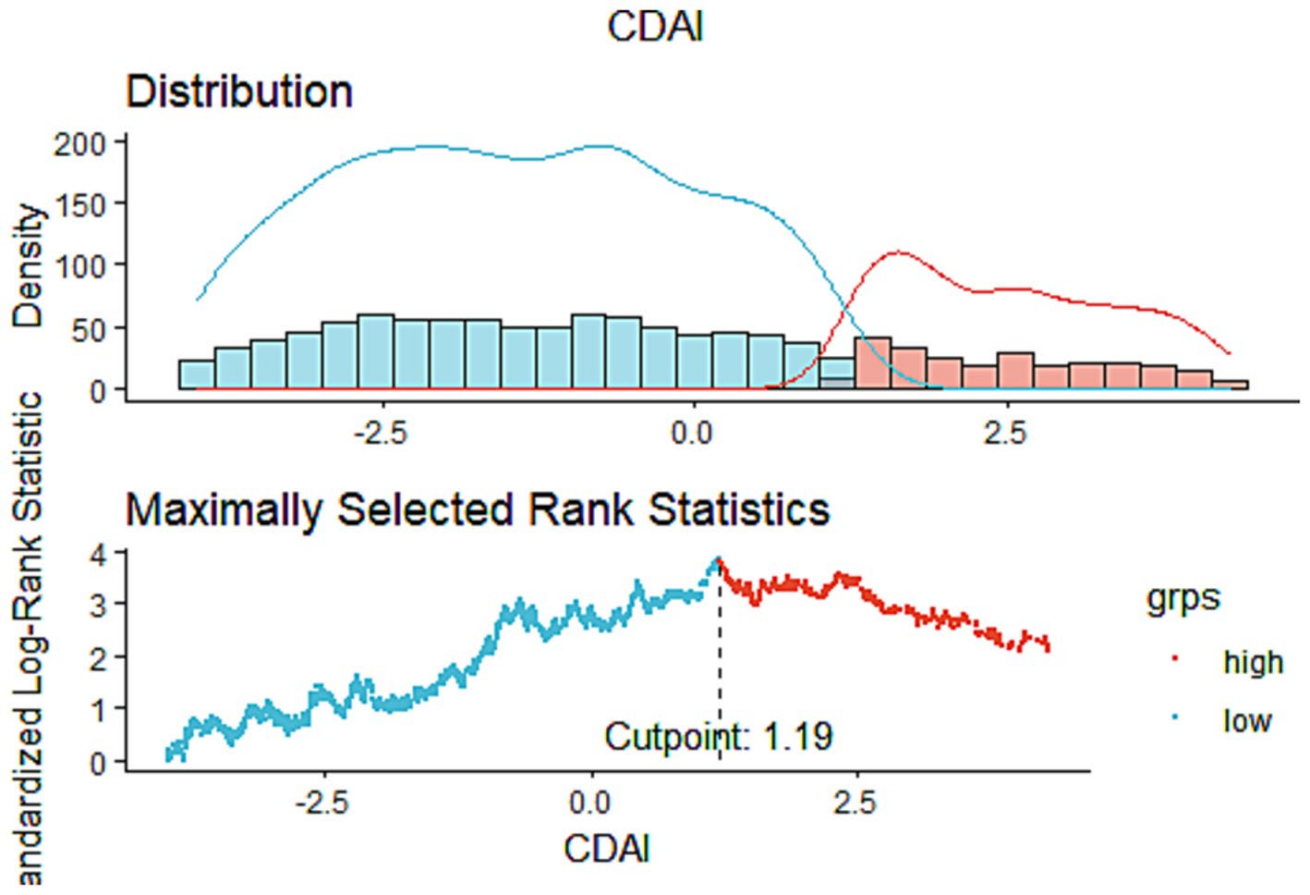
The cutoff point was calculated using the maximally selected rank statistics based on the “maxstat” package.

This study comprised 1,534 COPD patients in total. According to Table 1, white males aged 40–64 who have a high level of education, smoke, consume alcohol, and have hypertension are more likely to develop COPD. The optimal CDAI cutoff value (1.19) was determined according to MSRSM (Figure 2), and participants were divided into low CDAI group (Q1: CDAI <1.19) and high CDAI group (Q2: CDAI ≥1.19). Patients with low CDAI levels had more COPD deaths than those with high CDAI levels, suggesting that CDAI may be linked to the mortality risk in COPD patients.

**Table 1:** Characteristic of participants.

Variable	Total	CDAI		*p* value
		Q1	Q2	
Sex, *n* (%)				0.48
Female	664 (49.00)	488 (49.99)	176 (46.94)	
Male	870 (51.00)	624 (50.01)	246 (53.06)	
Age, years, *n* (%)				0.51
40–64	753 (58.85)	539 (58.01)	214 (60.62)	
≥65	781 (41.15)	573 (41.99)	208 (39.38)	
Race, *n* (%)				0.06
White	1,030 (84.19)	732 (82.81)	298 (87.10)	
Others	504 (15.81)	380 (17.19)	124 (12.90)	
Educational level, *n* (%)				< 0.001
< High school	486 (22.78)	392 (27.10)	94 (13.71)	
High school diploma	376 (25.38)	263 (23.41)	113 (29.54)	
> High school	672 (51.84)	457 (49.49)	215 (56.76)	
BMI, kg/m^2^, *n* (%)				0.78
< 25	428 (26.40)	309 (25.93)	119 (27.40)	
25–30	517 (33.49)	374 (33.04)	143 (34.43)	
> 30	589 (40.11)	429 (41.03)	160 (38.17)	
PIR, *n* (%)				< 0.001
< 1.3	548 (26.14)	423 (29.05)	125 (20.01)	
1.3–3.5	584 (36.60)	431 (38.46)	153 (32.71)	
> 3.5	402 (37.26)	258 (32.50)	144 (47.27)	
Health insurance, *n* (%)				0.86
No	143 (9.36)	108 (9.50)	35 (9.08)	
Yes	1,391 (90.64)	1,004 (90.50)	387 (90.92)	
Smoking status, *n* (%)				0.14
Former	759 (48.19)	542 (45.83)	217 (53.16)	
Never	241 (17.38)	174 (17.55)	67 (17.01)	
Now	534 (34.43)	396 (36.62)	138 (29.83)	
Alcohol consumption status, *n* (%)				0.01
No	112 (5.95)	92 (7.15)	20 (3.43)	
Yes	1,422 (94.05)	1,020 (92.85)	402 (96.57)	
Diabetes, *n* (%)				0.33
Borderline	53 (3.15)	36 (2.68)	17 (4.14)	
No	1,155 (79.35)	826 (78.68)	329 (80.76)	
Yes	326 (17.50)	250 (18.64)	76 (15.10)	
Hypertension, *n* (%)				0.26
No	650 (48.29)	474 (46.80)	176 (51.42)	
Yes	884 (51.71)	638 (53.20)	246 (48.58)	
COPD, *n* (%)				< 0.0001
Survival	932 (70.15)	642 (66.03)	290 (78.82)	
Death	602 (29.85)	470 (33.97)	132 (21.18)	

The *p*-value was calculated by the weighted linear regression model. % for categorical variables; Q1: CDAI < 1.19, Q2: CDAI ≥ 1.19. BMI, body mass index; PIR, poverty income ratio; CDAI, composite dietary antioxidant index.

### 3.2 Association of CDAI with all-cause mortality in COPD

During a median follow-up period of 92 months, 602 out of the 1,534 COPD patients died of all-cause mortality. In Model 1, we observed that for each unit increase in CDAI, the risk of all-cause mortality decreased by 7% (*HR* = 0.93, 95% *CI*: 0.91–0.96). After adjusting for multiple factors, each additional unit increase in CDAI was associated with a 5% reduction in all-cause mortality risk (Model 2, *HR* = 0.95, 95% *CI*: 0.92–0.98; Model 3, *HR* = 0.95, 95% *CI*: 0.92–0.97) (Table 2). When CDAI was categorized into high CDAI group and low CDAI group, Model 1, we found that COPD patients in the high CDAI group had a 41% lower risk of all-cause mortality compared to those in the low CDAI group (*HR* = 0.59, 95% *CI*: 0.46–0.77). The association between high CDAI and lower mortality risk remained in Models 2 and 3 even after controlling for several variables. COPD patients in the high CDAI group had a 35% lower probability of dying from all causes than those in the low CDAI group (Model 2: *HR* = 0.65, 95% *CI*: 0.50–0.85; Model 3: *HR* = 0.65, 95% *CI*: 0.50–0.85) (Table 2).

**Table 2:** The relationships between CDAI and all-cause mortality in COPD.

	Model 1		Model 2		Model 3	
	OR (95% CI)	*P*	OR (95% CI)	*P*	OR (95% CI)	*P*
All-cause mortality						
CDAI	0.93 (0.91,0.96)	<0.0001	0.95 (0.92,0.98)	<0.001	0.95 (0.92,0.97)	<0.001
CDAI category						
Q1	Ref		Ref		Ref	
Q2	0.59 (0.46,0.77)	<0.0001	0.65 (0.50,0.85)	0.002	0.65 (0.49,0.85)	0.002

Data were present as odds ratio (OR) and 95% confidence interval (95%CI). Model 1, Sex, age, and race were adjusted. Model 2, Sex, age, race, education, smoking, PIR, insurance, and drinking were adjusted. Model 3, Sex, age, race, education, smoking, PIR, insurance, drinking, BMI, diabetes, and hypertension were adjusted. Ref, reference; PIR, poverty income ratio; BMI, body mass index; CDAI, composite dietary antioxidant index. Q1: CDAI < 1.19, Q2: CDAI ≥ 1.19.

**Figure 3:**
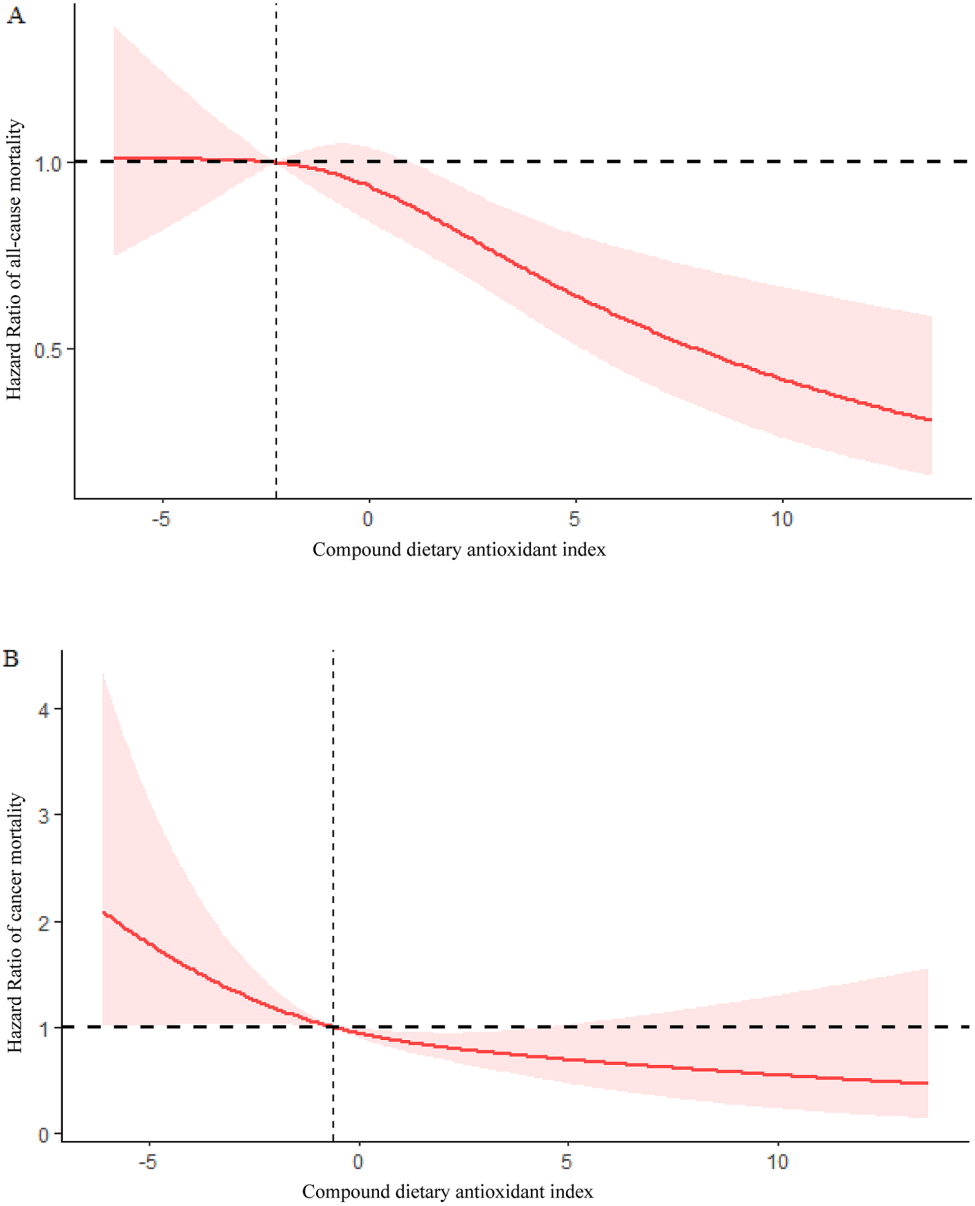
**(A)** Association between CDAI and risk of COPD all-cause mortality in the 1999–2018 NHANES survey. **(B)** Association between CDAI and risk of COPD cancer mortality in the 1999–2018 NHANES survey.

Using restricted cubic splines (RCS), it was found that CDAI and mortality from all causes were linearly associated (*P* for overall = 0.0007, *P* for non-linearity =0.1559) (Figure 3A).

Through subgroup analyses based on age, sex, race, BMI, and smoking status, we further investigated the relationship between CDAI values and all-cause mortality in individuals with COPD. The findings showed no significant interactions across subgroups and that the association between CDAI and all-cause mortality among COPD patients remained constant (*p* > 0.05) (Table 3).

**Table 3:** Subgroup analysis of CDAI and all-cause mortality in COPD.

Character	Q1	Q2	*P*	*P* for interaction
Sex, *n* (%)				0.07
Female	Ref	0.78 (0.52,1.16)	0.22	
Male	Ref	0.44 (0.30,0.66)	<0.0001	
Age, years, *n* (%)				0.81
40–64	Ref	0.63 (0.36,1.08)	0.09	
≥65	Ref	0.58 (0.42,0.81)	0.001	
Race, *n* (%)				0.09
White	Ref	0.53 (0.39,0.71)	<0.0001	
Others	Ref	1.00 (0.56,1.79)	1	
BMI, kg/m^2^, *n* (%)				0.23
< 25	Ref	0.57 (0.36,0.92)	0.02	
25–30	Ref	0.40 (0.23,0.68)	<0.001	
> 30	Ref	0.76 (0.47,1.24)	0.28	
Smoking status, *n* (%)				0.06
Former	Ref	0.43 (0.30,0.62)	<0.0001	
Never	Ref	0.58 (0.28,1.21)	0.15	
Now	Ref	0.87 (0.54,1.40)	0.57	

BMI, body mass index; 95% CI, 95% confidence interval; OR, odds ratio; Ref: Reference.

### 3.3 Association of CDAI with cancer mortality in COPD

A total of 1,083 COPD patients were included in the analysis of the association between CDAI and cancer mortality, with 151 patients dying from cancer. Further Cox regression analysis also indicated a significant association between CDAI and cancer mortality. In Model 1, we observed that for each unit increase in CDAI, the risk of cancer mortality decreased by 9% (*HR* = 0.91, 95% *CI*: 0.84–0.98). Following additional correction for several variables, every unit rise in CDAI was linked to an 8% (Model 2: *HR* = 0.92, 95% *CI*: 0.85–0.99) and 9% (Model 3: *HR* = 0.91, 95% *CI*: 0.85–0.98) reduction in cancer mortality risk (Table 4). Model 1 showed that the high CDAI group had a 63% lower risk of cancer mortality when CDAI was handled as a categorical variable (*HR* = 0.37, 95% *CI*: 0.22–0.63) compared to the low CDAI group. In Model 2, these statistical associations remained significant (*HR* = 0.41, 95% *CI*: 0.24–0.70). In Model 3, the relationship between high CDAI and reduced mortality risk persisted, with cancer mortality risk in the high CDAI group being reduced by 61% compared to the low CDAI group (*HR* = 0.39, 95% *CI*: 0.23–0.68) (Table 4).

**Table 4:** The relationships between CDAI and cancer mortality in COPD.

	Model 1		Model 2		Model 3	
	OR (95% CI)	*P*	OR (95% CI)	*P*	OR (95% CI)	*P*
Cancer mortality						
CDAI	0.91 (0.84,0.98)	0.01	0.92 (0.85,0.99)	0.03	0.91 (0.85,0.98)	0.01
CDAI category						
Q1	Ref		Ref		Ref	
Q2	0.37 (0.22,0.63)	<0.001	0.41 (0.24,0.70)	0.001	0.39 (0.23,0.68)	<0.001

Data were present as odds ratio (OR) and 95% confidence interval (95%CI). Model 1, Sex, age, and race were adjusted. Model 2, Sex, age, race, education, smoking, PIR, insurance, and drinking were adjusted. Model 3, Sex, age, race, education, smoking, PIR, insurance, drinking, BMI, diabetes, and hypertension were adjusted. Ref, reference; PIR, poverty income ratio; BMI, body mass index; CDAI, composite dietary antioxidant index. Q1: CDAI < 1.19, Q2: CDAI ≥ 1.19.

Based on RCS analysis, CDAI and cancer mortality were linearly associated in COPD patients (*P* for overall = 0.0145, *P* for non-linearity = 0.3948) (Figure 3B).

Furthermore, subgroup analyses by age, sex, race, BMI, and smoking status showed no significant interactions between subgroups and that the association between CDAI and cancer mortality in COPD patients remained constant (*p* > 0.05) (Table 5).

**Table 5:** Subgroup analysis of CDAI and cancer mortality in COPD.

Character	Q1	Q2	*P*	*P* for interaction
Sex, *n* (%)				0.28
Female	Ref	0.58 (0.24,1.39)	0.22	
Male	Ref	0.30 (0.16,0.56)	<0.001	
Age, years, *n* (%)				0.87
40–64	Ref	0.36 (0.15,0.84)	0.02	
≥65	Ref	0.36 (0.15,0.84)	0.02	
Race, *n* (%)				0.27
White	Ref	0.36 (0.20,0.66)	<0.001	
Others	Ref	0.72 (0.32,1.61)	0.42	
BMI, kg/m^2^, *n* (%)				0.16
< 25	Ref	0.55 (0.23,1.31)	0.17	
25–30	Ref	0.16 (0.06,0.42)	<0.001	
> 30	Ref	0.47(0.21,1.04)	0.06	
Smoking status, *n* (%)				0.52
Former	Ref	0.29 (0.15,0.59)	<0.001	
Never	Ref	0.55 (0.26,1.17)	0.12	
Now	Ref	0.42 (0.07,2.54)	0.35	

BMI, body mass index; 95% CI, 95% confidence interval; OR, odds ratio; Ref: Reference.

### 3.4 Sensitivity analyses

In the sensitivity analyses, after excluding COPD patients who died within two years of the start of follow-up, the results regarding the associations between CDAI and both all-cause mortality and cancer mortality in COPD patients remained unchanged (Table 6).

**Table 6:** Sensitivity analysis of CDAI and all-cause and cancer mortality in COPD.

	Model 1		Model 2		Model 3	
	OR (95% CI)	*P*	OR (95% CI)	*P*	OR (95% CI)	*P*
All-cause mortality						
CDAI	0.94 (0.89,0.98)	0.004	0.94(0.90,0.98)	0.01	0.93 (0.89,0.98)	0.003
CDAI category						
Q1	Ref		Ref		Ref	
Q2	0.61 (0.42,0.90)	0.01	0.63 (0.43,0.92)	0.02	0.62 (0.42,0.92)	0.02
Cancer mortality						
CDAI	0.87 (0.78,0.97)	0.01	0.85 (0.74, 0.96)	0.01	0.83 (0.73, 0.95)	0.01
CDAI category						
Q1	Ref		Ref		Ref	
Q2	0.22 (0.08,0.56)	0.002	0.17 (0.06, 0.49)	0.001	0.16 (0.05, 0.48)	0.001

Data were present as odds ratio (OR) and 95% confidence interval (95%CI). Model 1, Sex, age, and race were adjusted. Model 2, Sex, age, race, education, smoking, PIR, insurance, and drinking were adjusted. Model 3, Sex, age, race, education, smoking, PIR, insurance, drinking, BMI, Diabetes, and Hypertension were adjusted. Ref, reference; PIR, poverty income ratio; BMI, body mass index; CDAI, composite dietary antioxidant index. Q1: CDAI < 1.19, Q2: CDAI ≥ 1.19.

## 4 Discussion

Data from the NHANES were analyzed from 1999 to 2018 to examine the potential associations between CDAI and both all-cause and cancer mortality in US adults with COPD over the age of 40. We discovered that, in this cohort, CDAI was negatively correlated with the chances of both cancer and all-cause mortality after controlling for all confounding variables. Patients with COPD who scored higher on the CDAI were less likely to die from cancer and all causes. Additionally, stratified analyses revealed no variables that significantly affected the outcomes, suggesting that the association between CDAI and cancer and all-cause death is constant across subgroups. These findings confirm CDAI’s potential predictive role as a predictor of mortality in COPD patients.

As an innovative multidimensional tool, the CDAI systematically integrates concentrations of redox-active nutrients including water-soluble vitamins, trace elements, and polyphenolic compounds, thereby enabling standardized assessment of total dietary antioxidant defense capacity across populations (7). This concept is supported by numerous studies that have explored the relationship between dietary antioxidants and health outcomes. CDAI is a valuable tool for evaluating the relationship between a diet rich in antioxidants and various diseases. Studies have shown that a higher CDAI is associated with a reduced risk of developing multiple diseases, including hypertension, cancer, and obesity (16). The index assesses the intake of multiple dietary antioxidants, providing a comprehensive measure of antioxidant consumption and its potential health benefits. These studies suggest that an increase in the CDAI score is associated with a reduced likelihood of certain diseases. For instance, one study showed a favorable correlation between forced expiratory volume in one second (FEV1) and vitamin C, vitamin E, and total carotenoids (8). This indicates that CDAI may lower the risk of COPD by improving lung function (17). Another cross-sectional study observed that higher CDAI values are linked to a lower risk of all-cause and cancer-specific mortality in cancer survivors (3). According to our findings, modulating the CDAI can reduce COPD patients’ mortality due to all causes and cancer-specific causes. The association between antioxidant-rich diets and COPD remains robust even when age, sex, smoking status, and ethnicity are taken into account. This suggests the possibility of using antioxidant-rich diets as a primary prevention strategy. While our study primarily investigated dietary antioxidants in COPD progression, it is important to acknowledge the potential interplay of other modifiable lifestyle factors. Previous studies have demonstrated that physical activity levels are independently associated with reduced COPD exacerbations, and adherence to inhaled medications significantly improves lung function decline (18). Additionally, overall diet quality may exert synergistic effects beyond isolated antioxidants (19). The mechanism by which CDAI reduces the risk of COPD death remains unclear. One possibility is that circulating antioxidants may have direct effects on lung function. Alternatively, dietary antioxidants might lower COPD risk by reducing lung inflammation through the modulation of inflammatory markers like alkaline phosphatase and C-reactive protein (20).

Oxidative stress refers to a condition characterized by an elevated level of exogenous reactive oxygen and nitrogen species within the organism, leading to indiscriminate reactions with cellular components and resulting in oxidative damage, including DNA strand breaks, protein denaturation, and lipid peroxidation (21). This imbalance disrupts the homeostasis of the antioxidant defense system in the body, thereby triggering or accelerating inflammatory processes. Pro-inflammatory cytokines, reactive oxygen species, and oxidatively modified molecules produced during the inflammatory response further exacerbate oxidative stress, creating a vicious cycle (22). The body needs antioxidants to keep the balance between harmful reactive oxygen species and beneficial antioxidants. Research suggests that increasing antioxidant intake through diet can lower the prevalence of respiratory conditions (23). Additionally, oxidative stress-related inflammation and a reduction in endogenous antioxidant enzymes are also important mechanisms in the pathogenesis of certain malignancies (24). Multiple studies have shown that a diet rich in antioxidants has significant preventive and alleviating effects on COPD (25). This diet improves lung function by reducing the generation of free radicals and mitigating inflammation associated with oxidative stress, thereby lowering the incidence of COPD and slowing its progression. Antioxidants can neutralize reactive oxygen species in the body, inhibit oxidative damage, and maintain the structure and function of cells.

The pathogenesis of COPD is complex, and the role of CDAI in systemic inflammation may influence the risk of COPD through multiple mechanisms. Dietary fiber regulates the gut microbiome, promoting the production of anti-inflammatory metabolic products such as short-chain fatty acids, which help alleviate systemic inflammation (26). Additionally, dietary fiber can enhance immune function, improve resistance to oxidative stress, reduce the incidence of pulmonary inflammation, and regulate glucose and lipid metabolism, thereby decreasing the risk of metabolic diseases associated with COPD (27). Experimental evidence suggests that antioxidants modulate NF-κB signaling—a key pathway driving pro-inflammatory cytokine production in COPD lungs (28). By suppressing NF-κB activation, antioxidants may reduce neutrophil infiltration and mucus hypersecretion (29). In addition, animal models demonstrate that dietary antioxidants accelerate alveolar repair by promoting epithelial cell proliferation and inhibiting fibroblast-to-myofibroblast transition (30). These mechanisms collectively suggest that systemic antioxidants exert both protective and regenerative effects on lung tissue. The inverse association between dietary fiber intake and the risk of COPD mortality can be explained by these processes taken together, underscoring the potential benefit of nutritional interventions in lowering the risk of COPD-related death. Research has shown that higher dietary fiber intake is linked to lower levels of pro-inflammatory mediators, such as IL-6 and CRP (31). Furthermore, by controlling the innate immune system through the gut-liver-lung axis, dietary fiber may lower the risk of COPD (32). Vitamins A and *α*-tocopherol strengthen the antioxidant qualities of vitamins A, C, and E by reducing lipid peroxidation and neutralizing free radicals (33). A cross-sectional study found that adequate intake of vitamin A can delay the progression of COPD by improving lung function, thereby enhancing patients’ quality of life and reducing mortality rates (34). Similar to vitamin A, vitamin C maintains epithelial barrier integrity and affects immune cell function, thereby modulating cellular redox reactions (35). An experimental study on lung repair after tobacco-induced emphysema examined the impact of vitamin C supplementation in mice. Compared to the control group, the vitamin C supplementation group showed reduced activities of catalase, superoxide dismutase, and matrix metalloproteinases, along with lower levels of tumor necrosis factor-*α*, suggesting less lung tissue damage (36). Additionally, some studies have found a close association between vitamin C levels and all-cause mortality in patients with COPD (37). Additionally, Luo et al. found that vitamin C may serve as a protective factor against malignancies, including lung cancer. An increase of 100 mg/day in daily vitamin C intake correlates with a 7% decrease in the likelihood of lung cancer development (38). Vitamin E helps prevent lipid peroxidation. Studies have shown that increasing vitamin E intake is associated with a reduced prevalence of COPD (39). This data is consistent with a large cross-sectional investigation by Liu et al. that found a negative relationship between the incidence of COPD and vitamin E intake (40). The mechanism by which vitamin E reduces the risk of COPD is not fully understood. However, it is hypothesized that vitamin E may negatively regulate the epidermal growth factor receptor/mitogen-activated protein kinase (EGFR/MAPK) axis and inhibit cyclooxygenase 2 (COX2)-mediated nuclear translocation of phosphorylated STAT3. This regulation may help alleviate inflammation, apoptosis, and ROS in lung tissue caused by cigarette smoking, ultimately reducing the risk of acute exacerbation of COPD (41). Furthermore, intracellular antioxidant enzymes require zinc and selenium as cofactors. The respiratory system is supported by their ability to reduce inflammation and promote tissue repair (42). There is a link between the deficiency of these minerals and impaired respiratory function, as well as an increased chance of respiratory infection. Carotenoids, antioxidant pigments found in various foods, have been shown to protect the respiratory system by reducing the risk of asthma and COPD (43). Astaxanthin (AXT) is a natural carotenoid known for its antioxidant and anti-inflammatory properties. Research indicates that AXT can alleviate cigarette-induced emphysema through a SIRT1-dependent mechanism (44). After binding with SIRT1, AXT promotes the deacetylation of both SIRT1 and NRF2, leading to the production of antioxidant enzymes that reduce oxidative stress. Additionally, it inhibits the transcriptional activity of p65 NF-κB, thereby suppressing inflammatory responses (45). Another study has shown that AXT can improve the redox balance in cancer cells by targeting signaling molecules within tumor-associated signaling pathways, thereby inducing apoptosis and slowing the onset and progression of tumors (46). Therefore, increasing the intake of CDAI can help reduce all-cause mortality and cancer mortality in COPD patients.

This study shows notable strengths in several dimensions. First of all, this is the first time that the association between CDAI and the mortality rates from all causes and cancer in patients with COPD has been investigated using a large-scale, nationally representative dataset. Significantly, the results of the study are robust when based on a nationally representative sample for weighted analysis. Secondly, the sample size of this study is large and representative of the whole country.

Although our study has several limitations, these limitations should be considered carefully when interpreting and evaluating the results. Firstly, the NHANES database’s self-reported surveys serve as the main source of data, which could introduce memory bias and compromise data accuracy. Secondly, the process of sample selection excluded data with incomplete covariables, a practice that potentially omitted certain populations, thereby affecting the representativeness of the sample and the completeness of the analysis results. Moreover, although multivariate analysis was implemented to control for many known and potential confounding factors, there remain unaddressed potential residual confounders, particularly those that are difficult to measure. Given these limitations, future research should be based on large-scale longitudinal studies to enhance the validation of the findings. Furthermore, a more thorough knowledge of the mechanisms behind the association between CDAI and the mortality rates from all causes and cancer in individuals with COPD requires extensive basic study. Through these further studies, we can expect to obtain more solid scientific evidence, thereby contributing to more precise strategies for the prevention and management of chronic diseases.

## 5 Conclusion

In conclusion, we found that higher CDAI scores were negatively associated with all-cause mortality and cancer-specific mortality in patients with COPD.

## Data Availability

The original contributions presented in the study are included in the article/supplementary material, further inquiries can be directed to the corresponding authors.
